# Efficient Lightweight Multimodel Deep Fusion Based on ECG for Arrhythmia Classification

**DOI:** 10.3390/s22239347

**Published:** 2022-12-01

**Authors:** Mohamed Hammad, Souham Meshoul, Piotr Dziwiński, Paweł Pławiak, Ibrahim A. Elgendy

**Affiliations:** 1 Department of Information Technology, Faculty of Computers and Information, Menoufia University, Shibin El Kom 32511, Egypt; 2 Department of Information Technology, College of Computer and Information Sciences, Princess Nourah Bint Abdulrahman University, P.O. Box 84428, Riyadh 11671, Saudi Arabia; 3 Department of Intelligent Computer Systems, Czestochowa University of Technology, Armii Krajowej 36, 42-218 Czestochowa, Poland; 4 Department of Computer Science, Faculty of Computer Science and Telecommunications, Cracow University of Technology, Warszawska 24, 31-155 Krakow, Poland; 5 Institute of Theoretical and Applied Informatics, Polish Academy of Sciences, Baltycka 5, 44-100 Gliwice, Poland; 6 Department of Computer Science, Faculty of Computers and Information, Menoufia University, Shibin El Kom 32511, Egypt

**Keywords:** arrhythmia, CNN, ECG, lightweight, multimodel, fusion

## Abstract

An arrhythmia happens when the electrical signals that organize the heartbeat do not work accurately. Most cases of arrhythmias may increase the risk of stroke or cardiac arrest. As a result, early detection of arrhythmia reduces fatality rates. This research aims to provide a lightweight multimodel based on convolutional neural networks (CNNs) that can transfer knowledge from many lightweight deep learning models and decant it into one model to aid in the diagnosis of arrhythmia by using electrocardiogram (ECG) signals. Thus, we gained a multimodel able to classify arrhythmia from ECG signals. Our system’s effectiveness is examined by using a publicly accessible database and a comparison to the current methodologies for arrhythmia classification. The results we achieved by using our multimodel are better than those obtained by using a single model and better than most of the previous detection methods. It is worth mentioning that this model produced accurate classification results on small collection of data. Experts in this field can use this model as a guide to help them make decisions and save time.

## 1. Introduction

An arrhythmia is a series of abnormal heartbeats that tend to be electrical impulses that are too slow (bradyarrhythmia) or too fast (tachyarrhythmia), traveling through the heart very quickly through a faulty electrical circuit [1]. Individuals who have conditions including cardiomyopathy, hypertension, and coronary artery disease are more likely to have involvement in cardiac arrhythmias [2]. There are types of arrhythmias that are very dangerous and are not accompanied by any symptoms [3]. Arrhythmia can be diagnosed through an electrocardiogram (ECG). During this procedure, the electrical current involved in each heartbeat is graphically recorded so that it is easier for cardiologists to notice any abnormal patterns in the heartbeat and relate them to any abnormality that indicates it. ECG records consist of many distinctive waveforms, for example, the P wave (which helps to identify atrial contractions), the QRS complex (which tracks ventricular contractions), and the T wave (which is responsible for electrical activity). These waves are the principal features of any ECG signal, which helps in the diagnosis of various arrhythmias [4]. Artificial intelligence (AI) techniques have recently been used in a variety of medical applications [5,6,7,8] and especially in the classification of arrhythmias [9,10,11,12,13,14,15,16,17,18,19,20,21,22,23,24,25,26,27]. These AI techniques according to most of the previous works are classified into two main methods, such as feature-based approaches and deep learning approaches. The feature-based approaches have many limitations, such as suffering from overfitting, using complex models with a very high number of features and parameters, and obtaining low performance by using small data. To overcome the limitations of feature-based approaches, researchers introduced deep learning approaches. Deep learning obtains high accuracy by using big data and can overcome the overfitting problem [28]. Convolutional neural networks (CNNs), the most often employed deep learning method, have recently acquired prominence in the classification of arrhythmias by using ECG signals [9,10,11,12,13,14,15,16,17,18,19,20,21,22,23,24,25,26,27]. Automatic detection of this disease plays an important role in the immediate diagnosis of the heart. Therefore, in this study, we employed a convolutional neural network (CNN) for arrhythmia classification. However, previous deep learning approaches suffer from many limitations, such as obtaining low performance on small data, being time consuming, and being very complex with high parameters. In this research, we try to solve these difficulties by proposing our model.

Although there have been several deep learning methods for the classification of arrhythmias, we are going to propose a new lightweight multimodel deep fusion for the classification of arrhythmias in this paper. For this purpose, two lightweight deep learning models are employed and combined into one model for arrhythmia classification. We have used one of the most common heart disease datasets (the MIT–BIH dataset) to evaluate our model. The following are significant novel contributions of this research.

- We propose a lightweight deep learning model with low parameters and a small number of layers, which makes this model less complex than other previous models for arrhythmia classification. We proposed a model that consists of lower layers and achieved high accuracy, whereas other lightweight models obtained very low accuracy.
- We propose a novel multimodal deep fusion and hypered architecture that makes use of CNNs based on ECG for arrhythmia classification. Our model investigates whole signals rather than segments, which avoids unnecessary computations when segments overlap and allows our network to scale up more effectively as signal quality increases.
- The proposed multimodel is a totally end-to-end learning model that uses CNN for single-lead ECG signal, which decreases the complexity of the model and decreases the time of implementation. Unlike some previous models that used separate classifiers or separate stages for extracting or selecting the features, our model combines all these stages into only one stage.
- The cross-validation technique is employed to solve the unbalanced problem of the used data. In addition, it makes our system more robust and fix the overfitting problem.
- We demonstrate an accurate model that outperforms most of the previous methods on small datasets, especially the lightweight deep models.

## 2. Previous Methods for Arrythmia Classification

Arrhythmia classification using ECG signals is a crucial diagnostic technique for identifying cardiovascular disorders. Early arrhythmia classification methods focused on feature-based models or rule-based algorithms, and they have been changed to models with raw ECG data input or minimal modification [29,30,31]. Recently, arrhythmia-classification methods have focused on deep learning approaches, especially with big data [9,10,11,12,13,14,15,16,17,18,19,20,21,22,23,24,25,26,27]. We omitted the feature-based methods and confined to deep learning approaches in accordance with the scope of this work. In addition, almost all recent works focused on deep learning, according to [32,33].

Various methods have been introduced for arrhythmia classification using deep learning [9,12,13,15,16,17]. One of the earliest methods that used deep learning was presented by Kiranyaz et al. in [9], which implemented an adaptive 1-D CNN on the MIT–BIH database for the detection of two types of arrhythmias which gave performance classification with an accuracy of 97.6% for all records. Xu et al. [12] presented an end-to-end technique employing a deep neural network for extracting the features and classification based on heartbeat alignment, achieving a 99.70% total accuracy. In [13], Fan et al. detected atrial fibrillation by using a multiscaled deep CNN (MS-CNN) and achieved an overall accuracy of 96.99% and 98.13% on ECG recordings of 5 s and 30 s respectively. Amin Ullah et al. [15] presented a deep learning method to classify arrhythmia from 2D ECG images. They worked with the MIT–BIH database and reached a classification accuracy of 98.92% on average. Hammad et al. [16] presented a CNN and convolutional long short-term memory (ConvLSTM) model for arrhythmia detection, which can be utilized by Internet of things (IoT) applications. They obtained the highest accuracy of 98% by using the CNN model. Moreover, based on deep learning, Hammad et al. [17] suggested a multitiered model for arrhythmia identification. They used LSTM as a feature extractor and k-nearest neighbour (k-NN) as a separate classifier for classification. They obtained an average of 98% accuracy.

Diker et al. [10] introduced a deep method based on several transfer models for arrhythmia classification. They converted the ECG signals to 2D spectrograms images then fed the images to the deep models. They obtained the best accuracy of 83.82% by using AlexNet.

Singh et al. [11] employed recurrent neural network (RNN) for classifying the ECG signals as normal or arrhythmic. They obtained the highest accuracy of 88.1% when using RNN with LSTM.

Panda et al. [14] introduced a method based on FFREWT filter-bank and CNN approach for arrhythmia classification. They obtained an average accuracy of 97.592% using 8sec ECG signals.

Rahul and Sharma [34] used bidirectional LSTM for classifying ECG signals as normal or as one type of arrhythmia. They first convert each ECG signal to 2D images. After that, they performed the preprocessing stage on the images by using several filters and image-processing techniques. Finally, they fed the preprocessed images to the model for classification. They used 4sec ECG segment and obtained a better accuracy of 98.85%.

However, the majority of these previous works used small datasets with low accuracy and calculated the computational complexity of the work as in [9,11,12,13,14,16]. In addition, some papers suffer from overfitting problems and it is computationally intensive for them to learn the features [10,15,16,17]. Furthermore, the methods in [9,13,14] are not robust and obtain low performance with big and small data. Finally, all of these methods need a high processing time to implement their deep models.

A new and efficient lightweight multimodal approach for the classification of arrhythmia is presented in this paper to overcome the issues of the previous works. The proposed method outperforms the majority of existing algorithms on both small and large datasets. Additionally, compared to earlier low-resource deep learning methods for arrhythmia classification, our approach is more robust. The proposed multimodel is elaborated on in the subsequent section.

## 3. Materials and Methods

**Figure 1:**
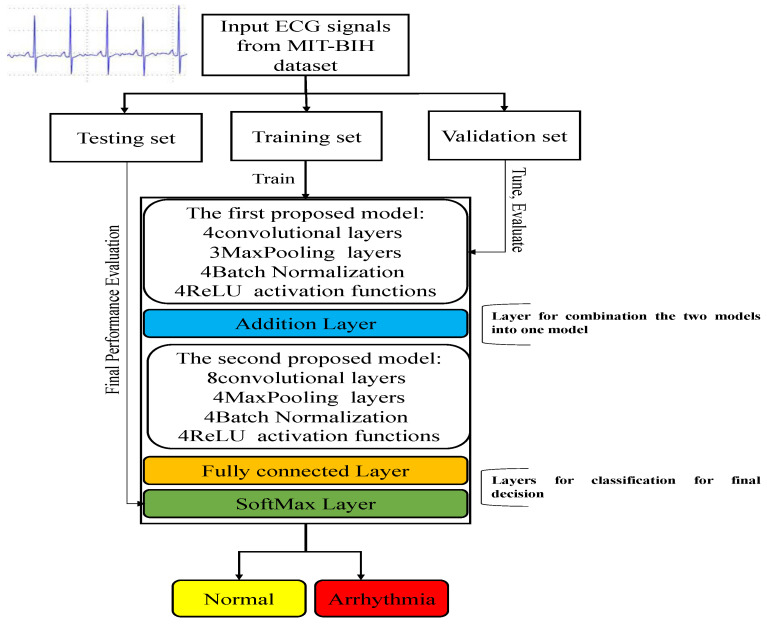
Block diagram of our multimodel deep fusion.

A novel methodology as shown by the block diagram in Figure 1 is built into this work to achieve the classification of arrhythmia. This methodology uses a hybrid model in which two lightweight CNN models are used for feature extraction and combined for classification. This method eliminates the handcrafted feature-extraction process that saves time and manpower and improves the percentage of automation in ailment classification. In addition, the input ECG signals are converted to 2D images and fed directly to our model without any preprocessing stages. We converted the 1D signals to 2D images to be suitable for our deep model and also to eliminate the signal noise [35]. Furthermore, the proposed model works on the whole signals without the need for segmentation or division, which avoids unnecessary computations when segments overlap.

### MIT–BIH Database

**Figure 2:**
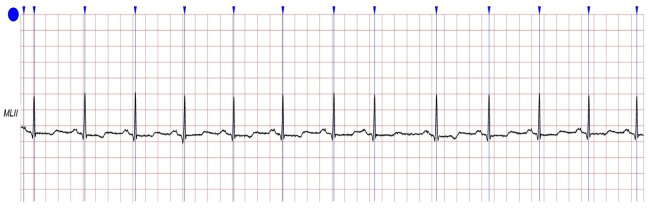
A typical example of an ECG signal from used database (MLII of record 100).

**Figure 3:**
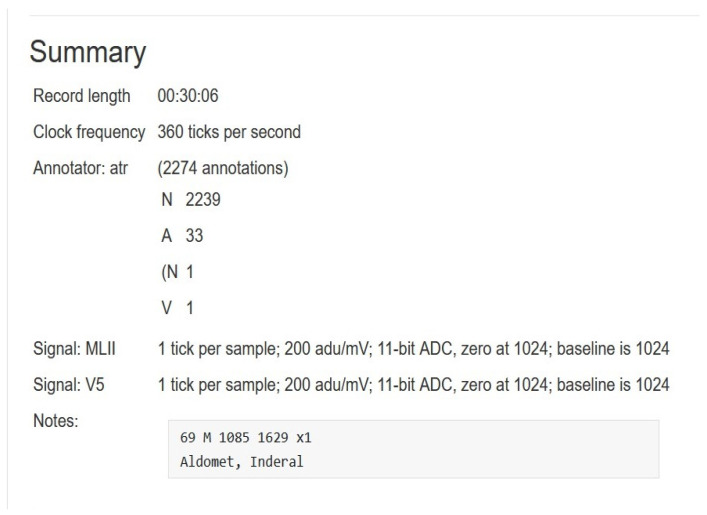
Summary of one record from MIT–BIH database.

The proposed method was performed on ECG signals of arrhythmia, which were considered from the most common database (MIT–BIH Arrhythmia Database [36,37]) that comprises 48 patient records which are digitized at 360 samples per second. The resolution of each signal is 11 bits over a 10-mV range. Each record is labeled by two or more cardiologists. The total uncompressed size of all files in this database is 104.3 MB, and the database is freely available for download at physionet.org. The records in the data are numbered as follows: from 100, the first record, to 109, from 111 to 124 (except record number 120, not included), and from 200 to 234, the last record (except records 204, 206, 211, 216, 218, 224, 225, 226, 227 and 229, not included). In the majority of medical records, the upper signal is a modified limb lead II (MLII), which is produced by putting electrodes on the chest. Typically, the lower signal is a modified V1 lead (occasionally V2 or V5, and in one instance V4). In this paper, we worked only on MLII because all the needed information is in this lead and to reduce the complexity of the system. Figure 2 shows a visualized example for lead MLII of one record (record 100) from the database. In addition, Figure 3 shows the summary of the record (record 100).

In the related work section, we discovered that every deep learning-based research effort is based on a single model or a comparison of multiple models. In this section, we provide a full explanation of our proposed models, which integrate two models and take advantage of the unique qualities of each.

Moreover, we test three cases based on the used dataset and these cases are as follows.

**Figure 4:**
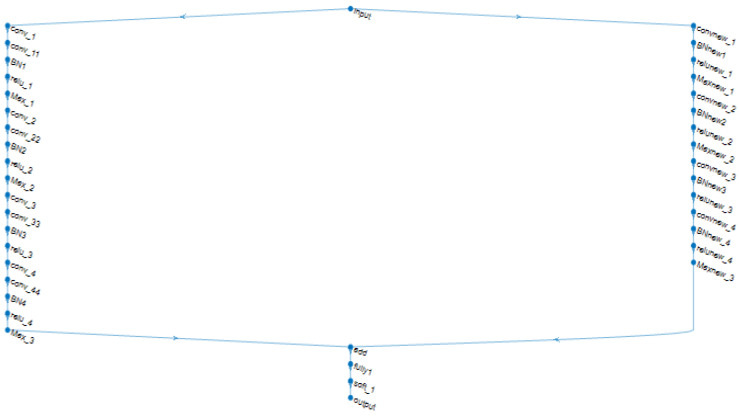
The architecture of our multi-modal.

1. In the first case, we tested the proposed first deep model as a single model and evaluated the performance of this model on the dataset. This model consists of four convolutional layers and each one is followed by one batch normalization, maxpooling layer, and ReLU activation function. Table 1 shows the analysis of the layers for the first proposed model.
2. In the second case, we tested the proposed second deep model as a single model and evaluated the performance of this model on the dataset. This model consists of eight convolutional layers and every two layers are followed by one batch normalization, maxpooling layer, and finally ReLU activation function. Table 2 shows the analysis of the layers for the second proposed model.
3. In the third case, we combine the two models into one model to take advantage of their prior knowledge and weights found for arrhythmia classification. Figure 4 shows the architecture of our multimodal where the input is the ECG signals from the dataset.

In the first case, we build our first model by using 14 layers, for which the input size of the ECG 2D image is 400 × 400 × 3. The first layer we added is a convolutional layer to extract high-level features from the input images with a stride of 1 × 1 and a small filter size of 3 × 3 for better generalization. We employed the padding = ’same’ to ensure that the filter is applied to all elements of the input images. The second layer is a batch normalization to solve the internal covariate shift problem, and by which we can use a higher learning rate and train the model faster. After that, we add an activation function to decide which feature can be activated and transferred to the next layer. In our model, we employed ReLU as an activation function, as it does not activate all the features at the same time, which takes less time compared to other activation functions such as the sigmoid function. Next, we added a 2D maxpooling layer with stride 2 × 2 and padding = [0 0 0 0]. This layer helps in reducing the dimension of the feature map. As a result, we reduce the number of parameters and the amount of computation in our model. The output of the maxpooling is an image with a size of 200 × 200. We repeated the previous layers one round more in the same order and with the same number, which is a convolutional layer with stride 1 × 1 and padding = ’same’, batch normalization layer, ReLU activation function followed by a maxpooling layer with stride 2 × 2 and padding = [0 0 0 0]. The output of this maxpooling is a feature map with a size of 100 × 100. At this point, we repeated the previous layers except for the maxpooling layer. The output of the third convolution pass through normalization and the activation function is fed to the fully connected layer. In this layer, a 2D feature vector is transformed to a 1D feature vector for classification. The output of the fully linked layer is subsequently passed to the softmax layer for classification. This model has only three convolutional layers and one fully connected layer, making it a lightweight model.

In the second case, we built a model by using 21 layers for which the input size of the ECG 2D image is 400 × 400 × 3. We added almost the same number of layers as in the first case except for a few little changes as follows.

- Instead of using one convolutional layer, we used two convolutional layers, which allows a hierarchical decomposition of the input.
- We employed eight convolutional layers instead of using three convolutional layers, which makes the model deeper and capable of extracting more features.
- We employed in this model three maxpooling layers and the output of the third one was passed to the fully connected layer. In contrast to the first model, the output of the last convolutional layer is passed to the fully connected layer.
- The size of the feature map that is fed to the fully connected layer is 50 × 50, unlike the first model which is 100 × 100.
- Finally, this model consists of eight convolutional layers and only three maxpooling layers, which is considered to be a lightweight model.

In the case of multimodal, we combine the first model with the second model into one model. This combination achieved better performance than both systems. The combination is done by using the addition layer, which adds the output features of the layers from both models into the same block, which has been called the “add layer”. All inputs to an addition layer must have the same dimension. We used the following MATLAB function to build the add layer: layer = additionLayer(numInputs) creates an addition layer that adds numInputs inputs element-wise. The output layer is a sort of fully connected layer composed of the output rows’ neurons. Softmax is then used to make the final determination of the system whether the ECG signals are arrhythmic or normal.

## 4. Experimental Study, Results, and Discussion

### 4.1. Implementation Environment, Performance Metrics, and Evaluation Method

The models were run in MATLAB 2019b by using the deep learning toolbox, which provides a platform for developing and executing deep neural networks with algorithms, pretrained models, and applications. In the first part of this section, we evaluated all models (both single models and the proposed multimodal) on the used dataset. After that, in this section, the proposed model was compared to previous models in this area.

To validate our multimodal, accuracy (Accur), positive predictivity (+Pr), specificity (Speci), and sensitivity (Sensi) are chosen as measures for evaluation, which are defined as follows,

(1) $$Accuracy=\frac{TP+TN}{TP+FN+TN+FP}$$

(2) $$Specificity=\frac{TN}{TP+FP}$$

(3) $$Sensitivity=\frac{TP}{TP+FN}$$

(4) $$+Pr=\frac{TP}{TP+FP},$$

where *TP* is true positives, *FP* is false positives, *FN* is false negatives, and *TN* is true negatives.

In this paper, we employed cross-validation techniques to overcome the overfitting problem and the unbalanced problem of the data by dividing the whole data into K-fold [38]. This technique divides the data into two parts: one for learning or training and the other for testing or validation. According to “K”, the technique will divide the data. In this paper, we employed a tenfold technique, therefore, the data is divided into 10 equal parts (nine parts for learning and one part for testing). After that, the other nine parts are taken for training and another part for testing (by shifting the test part from one to the left). These steps are repeated until the whole dataset is used for training and testing and obtains accuracy at every step. Finally, we compute the average of all accuracies to be the final accuracy of our system (in our case, we computed the average of 10 accuracies).

### 4.2. Experimental Results and Discussion

**Figure 5:**
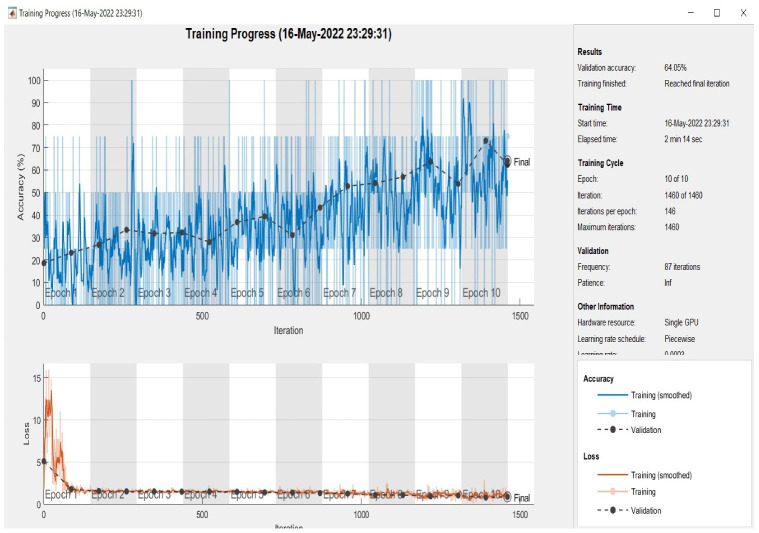
Accuracy in training and validation (the upper plots) and loss in training and validation (the bottom plots) for the first model (15 layers) over 10 epochs.

**Figure 6:**
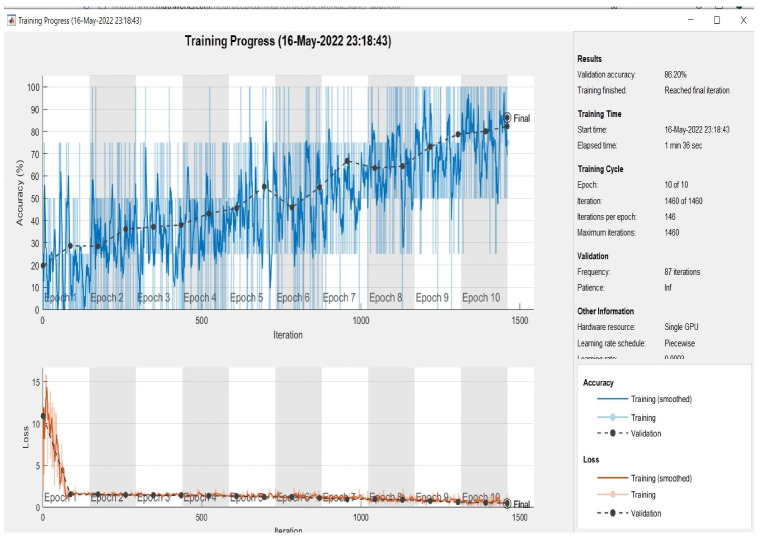
Accuracy in training and validation (the upper plots) and loss in training and validation (the bottom plots) for the second model (19 layers) during 10 epochs.

**Figure 7:**
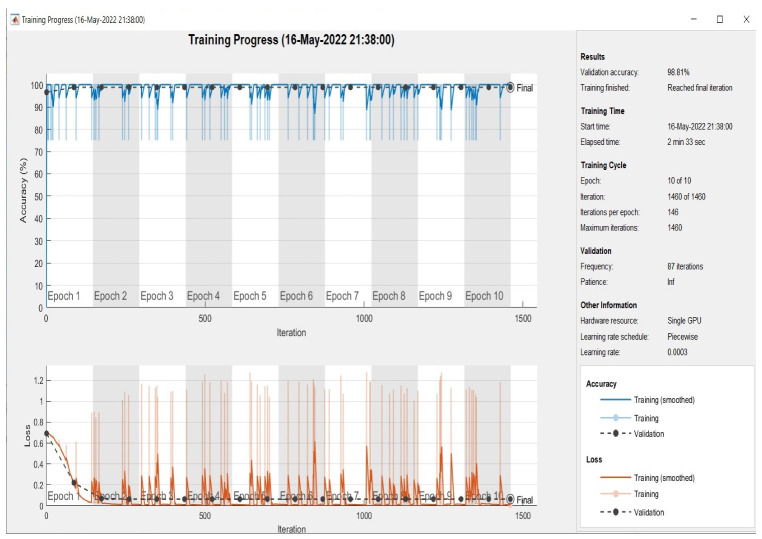
Accuracy in training and validation (the upper plots) and loss in training and validation (the bottom plots) for the proposed multimodel during 10 epochs.

At this point, all models were evaluated on the test set, which had been randomly selected from the dataset. As demonstrated in Figure 5, Figure 6 and Figure 7 the plotting of the training and the validation accuracy’s curves converge, indicating that the training has stabilized after seven epochs for the first and second models and after three epochs for the multimodal, and that the accuracy has increased.

The models are trained on the training samples and then evaluated on the test samples, which raises the values of the model’s final layer’s hidden coefficients to better fit the ECG images to be trained at each stage. This technique is repeated for each training epoch, and after epoch 9 for single models and epoch 5 for multimodel models, the accuracy does not improve beyond a specific threshold. This indicates that every time the models are trained on a set of ECG images from the training sample, they validate images from the validation sample and attain a particular level of accuracy.

Figure 5 illustrates that the first model’s accuracy during the training and validation phase was 64.05%. Figure 6 illustrates that the accuracy of the second model during training and validation was 86.20%. Finally, we can observe from Figure 7 that the proposed multimodel achieved an accuracy of 98.81% during the training and validation phase. The overall performance of all models based on the metrics is shown in Table 3.

From Table 3, we can show that the first model’s ability to predict the positive ECG signals from the dateset is 54.2%. In addition, from Table 3, we can find that the second model’s ability to predict the positive ECG signals is 85.60%. Finally, the same table shows that the proposed multimodal ability to predict the positive ECG signals is 98.80%. We can conclude from the results that the model with the smallest number of layers obtained low accuracy compared with the deeper models (in our case, the second model is the deeper). The deeper model extracts deep features and extracts more details than the small model, which increases the learning of the model to the input features, and as a result increases the accuracy. In addition, the first and second models are both lightweight models, which decrease the time and the complexity but with low accuracy. In this paper, we overcome this limitation by combining the two lightweight models into one lightweight multimodal with a high accuracy of 98.80% compared with other lightweight models (as both single models in our case). Table 4 shows the performance of the proposed multimodal in each fold by using the tenfold technique.

The arrhythmia classification system uses AI to assist physicians in determining if a patient is infected or not. This aids in lowering the occurrence of medical diagnosis errors and saves the doctor time and effort by providing faster and more accurate findings. Thus, it contributes to the reduction of heart disease-related deaths. Consequently, numerous investigations proposed various categorization methods for arrhythmia [9,10,11,12,13,14,15,16,17,34]. As shown in Table 5, a set of previously trained models with a small number of layers was examined, analysed, and compared to the proposed multimodel in terms of accuracy and other criteria [10,11,14,15,16,17,34].

From Table 5, we can observe that our model achieved high accuracy and the lowest elapsed time compared with most of the studies mentioned in the table. Huang et al. [10] obtained higher accuracy compared with our model (only 0.2%). However, this model has more layers with a higher elapsed time than the proposed model. Amin Ullah et al. [15] also obtained good accuracy but suffered from several limitations, such as the use of complex architecture, which led to an increase in the elapsed time of the model. In [12], they obtained higher accuracy than our method; however, they obtained low accuracy on small data. In addition, their method suffers from overfitting problems. For [13], they obtained lower results compared with our model. In addition, they used more layers and a more complex model compared with the proposed model. Other previous methods mentioned in the previous table [11,14,16,17] obtained lower accuracy than our method with higher implementation time.

Even though there have been numerous contributions to this subject, all of them have concentrated on the use of a single model to classify arrhythmia, and there has been no attempt to combine multiple models to use the unique qualities of each model. Furthermore, most previous works (particularly lightweight models) produced poor results with small data, whereas our multimodal approach produced better results with small data. We can conclude that the architecture of our model is unique due to the creation of a novel architecture able to exploit the strength of two CNN models.

## 5. Conclusions

This paper’s main contribution is to propose an efficient lightweight deep learning model for arrhythmia classification-based ECG signals. We proposed a new multimodel based on a combination of two lightweight models with a small number of layers and a small number of parameters, which achieved high accuracy compared with other previous models. The proposed model is fast, less complex than other previous models, and can be implemented for mobile applications. In this study, we overcome most of the common limitations of previous deep models by achieving high accuracy by using small data and by addressing unbalanced data problems, overfitting problems, and computational complexity problems. Our model achieved an accuracy of 98.80%, a specificity of 98.80%, and a sensitivity of 98.80%, which are acceptable results for medical applications. In the future, we can employ this model on more datasets to classify more heart cases. In addition, we can apply our model to other kinds of signals such as brain signals, and observe the performance and its effect on these kinds of signals. Finally, we can study the effect of using multilead ECG signals on the proposed model.

## Figures and Tables

**Table 1:** Analysis of the layers for our first model.

No.	Name	Type	Activation	Learnable
1	imageinput 400 × 400 × 3 images	Image Input	400 × 400 × 3	-
2	conv_1 8 3 × 3 × 3 convolutions with stride [1 1] and padding ’same’	Convolution	400 × 400 × 8	Weight 3 × 3 × 8 Bias 1 × 1 × 8
3	batchnorm_1 Batch normalization with 8 channels	Batch normalization	400 × 400 × 8	Offest 1 × 1 × 8 Scale 1 × 1 × 8
4	relu_1 ReLU	ReLU	400 × 400 × 8	-
5	maxpool_1 2 × 2 max pooling with stride [2 2] and padding [0 0 0 0]	Maxpooling	200 × 200 × 8	-
6	conv_2 16 3 × 3 × 8 convolutions with stride [1 1] and padding ’same’	Convolution	200 × 200 × 16	Weight 3 × 3 × 8 × 16 Bias 1 × 1 × 16
7	batchnorm_2 Batch normalization with 16 channels	Batch normalization	200 × 200 × 16	Offest 1 × 1 × 16 Scale 1 × 1 × 16
8	relu_2 ReLU	ReLU	200 × 200 × 16	-
9	maxpool_2 2 × 2 max pooling with stride [2 2] and padding [0 0 0 0]	Max Pooling	100 × 100 × 16	-
10	conv_3 32 3 × 3 × 16 convolutions with stride [1 1] and padding ’same’	Convolution	100 × 100 × 32	Weight 3 × 3 × 16 × 32 Bias 1 × 1 × 32
11	batchnorm_3 Batch normalization with 32 channels	Batch normalization	100 × 100 × 32	Offest 1 × 1 × 32 Scale 1 × 1 × 32
12	relu_3 ReLU	ReLU	100 × 100 × 32	-
13	fc 5 fully connected layer	Fully Connected	1 × 1 × 5	Weight 5 × 320,000 Bias 5 × 1
14	softmax softmax	softmax	1 × 1 × 5	-
15	classoutput crossentropyex	Classification Output	-	-

**Table 2:** The analysis of the layers for the second proposed model.

No.	Name	Type	Activation	Learnable
1	inputimage 400 × 400 × 3 images	Input Image	400 × 400 × 3	-
2	convol_1 8 3 × 3 × 3 with stride = [1 1] and padding = ’same’	Convolutional	400 × 400 × 8	Weight 3 × 3 × 8 Bias 1 × 1 × 8
3	convol_2 8 3 × 3 × 8	Convolutional	400 × 400 × 8	Weight 3 × 3 × 8 × 8 Bias 1 × 1 × 8
4	batchnorm_1 with 8 channels	Batch normalization	400 × 400 × 8	Offset 1 × 18 Scale 1 × 1 × 8
5	relu_1	ReLU	400 × 400 × 8	-
6	maxpool_1 2 × 2 with stride = [2 2] and padding = [0 0 0 0]	Maxpooling	200 × 200 × 8	-
7	convol_3 16 3 × 3 × 8	Convolutional	200 × 200 × 16	Weight 3 × 3 × 8 × 16 Bias 1 × 1 × 16
8	convol_4 16 3 × 3 × 16	Convolutional	200 × 200 × 16	Weight 3 × 3 × 16 × 16 Bias 1 × 1 × 16
9	batchnorm_2 with 16 channels	Batch normalization	200 × 200 × 16	Offest 1 × 1 × 16 Scale 1 × 1 × 16
10	relu_2	ReLU	200 × 200 × 16	-
11	maxpool_2 2 × 2	Maxpooling	100 × 100 × 16	-
12	convol_5 32 3 × 3 × 16	Convolutional	100 × 100 × 32	Weight 3 × 3 × 16 × 32 Bias 1 × 1 × 32
13	convol_6 32 3 × 3 × 32	Convolutional	100 × 100 × 32	Weight 3 × 3 × 32 × 32 Bias 1 × 1 × 32
14	batchnorm_3 with 32 channels	Batch normalization	100 × 100 × 32	Offest 1 × 1 × 32 Scale 1 × 1 × 32
15	relu_3	ReLU	100 × 100 × 32	-
16	convol_7 64 3 × 3 × 32	Convolutional	100 × 100 × 64	Weight 3 × 3 × 32 × 64 Bias 1 × 1 × 64
17	convol_8 64 3 × 3 × 32	Convolutional	100 × 100 × 64	Weight 3 × 3 × 64 × 64 Bias 1 × 1 × 64
18	batchnorm_4 with 64 channels	Batch normalization	100 × 100 × 64	Offest 1 × 1 × 64 Scale 1 × 1 × 64
19	relu_4	ReLU	100 × 100 × 64	-
20	maxpool_3 2 × 2	Maxpooling	50 × 50 × 16	-
21	fc 5 fully connected layer	Fully Connected	1 × 1 × 5	Weight 5 × 160,000 Bias 5 × 1
22	softmax	Softmax	1 × 1 × 5	-
23	classoutput crossentropyex	Classification Output	-	-

**Table 3:** Performance of both single models compared with our multimodel.

Model	Accuracy (Acc)	Speciificity (Speci)	Sensitivity (Se)	Elapsed Time
First Model	0.640	0.761	0.542	96 s
Second Model	0.862	0.897	0.826	134 s
Multimodal	0.988	0.988	0.988	153 s

**Table 4:** Performance of the proposed multimodal for each fold.

Fold#	TP	FN	FB	TN	+Pr	Se	Acc
1	42	1	0	5	1	0.976	0.979
2	42	1	1	4	0.976	0.976	0.958
3	43	0	0	5	1	1	1
4	43	0	0	5	1	1	1
5	43	0	2	3	0.955	1	0.961
6	43	0	0	5	1	1	1
7	41	2	0	5	1	0.953	0.980
8	43	0	0	5	1	1	1
9	43	0	0	5	1	1	1
10	41	2	1	4	0.976	0.953	0.997
Avg/total	424	6	4	46	0.990	0.986	0.988

**Table 5:** Comparison between previous models with low layer numbers and our multimodal.

Author Ref.	Methodology	No. of Layers	Elapsed Time	Performance
Diker et al. [10]	CNN	12	N/A	Acc = 0.8382 Se = 0.9545 Speci = 0.6250
Singh et al. [11]	RNN+LSTM	10	N/A	Acc = 0.8810 Se = 0.9240 Speci = 0.8335
Xu et al. [12]	DNN	10	N/A	Acc = 0.9970 Se = 0.9768 Speci = 0.9989
Fan et al. [13]	CNN	22	N/A	Acc = 0.9813 Se = 0.9377 Speci = 0.9877
Panda et al. [14]	CNN	11	2151.055 s	Acc = 0.9759, Speci = 0.9955%, Se = 0.9314
Rahul and sharma et al. [34]	Bi-directional LSTM	102	N/A	Acc = 0.9885, Speci = 0.9890%, Se = 0.9880
Amin Ullah et al. [15]	2D CNN	24	N/A	Avg Acc = 98.92%, Avg Speci = 99.67%, Avg Se = 97.26%
Hammad et al. [16]	CNN+ConvLSTM	35	7200 s	Best Acc = 98%
Hammad et al. [17]	LSTM+Genetic+KNN	38	322.35 s	Acc = 98.00%, Se = 99.70%, Speci = 95.80%
Proposed Model	Fusion of CNN	22	153 s	Acc = 98.80%, Se = 98.80%, Speci = 98.80%

## Data Availability

Not applicable.
